# Triphase contrast-enhanced CT to evaluate indications for autologous liver transplantation in patients with end-stage hepatic alveolar echinococcosis

**DOI:** 10.1038/s41598-021-01586-8

**Published:** 2021-11-11

**Authors:** Jing Chen, Li Wei, Tian-Wu Chen, Rui Li, Xiao-Ming Zhang, Chun-Mei Deng, Yi Zhang, Jing Xiong, Xian-Zhong Li, Zhi-Hong Li

**Affiliations:** 1 Sichuan Key Laboratory of Medical Imaging, and Department of Radiology, Affiliated Hospital of North Sichuan Medical College, 1# Maoyuan Road, Shunqing District, Nanchong, 637000 Sichuan China; 2 Department of Radiology, Ganzi Hospital, West China Hospital of Sichuan University (Ganzi Tibetan Autonomous Prefecture People’s Hospital), 94# Xida Road, Kangding, Ganzi, 626000 Sichuan China

**Keywords:** Parasitic infection, Diagnostic markers

## Abstract

Autologous liver transplantation (ALT) to cure end-stage hepatic alveolar echinococcosis (HAE) requires that hepatobiliary surgeons understand the invasion of intrahepatic structure and adjacent tissues or organs. Triphase contrast-enhanced CT of the liver has been widely used for diagnosis and preoperative evaluation of HAE. Three-dimensional (3D) reconstruction allows for accurate measurement of remnant liver volume (RLV). The objective of the study was to evaluate value of triphase contrast-enhanced CT together with 3D reconstruction in preoperative evaluation of indications for ALT in patients with end-stage HAE. This cohort include twenty-one consecutive patients with end-stage HAE, who preoperatively underwent triphase enhanced CT together with 3D reconstruction for ALT. To depict the indications, the 2D image data were reviewed statistically focusing on porta hepatis invasion, retrohepatic vena cava (RHVC) involvement and degrees of intrahepatic vessel invasion, and the 3D reconstruction was performed to obtain ratio of RLV to standard liver volume (SLV). The results showed that 95.24% patients (20/21) had porta hepatis invasion. When lesions located in right liver lobe, porta hepatis invasion occurred most commonly in the second and third porta hepatis (7/10), whereas the first, second and third porta hepatis were most commonly invaded by lesions in the right and caudate / left medial liver lobes (7/11) (*P* < 0.05). The mean value of longitudinal invasion of RHVC was 8.0 cm, and 95.2% (20/21) of patients had RHVC invasion with ≥ 180° circumferential invasion. As for the important vascular events, moderate and severe invasion occurred most commonly in the right hepatic vein, right branch of portal vein and RHVC each in 95.2% (20/21) patients (*P* < 0.05). We also found that preoperative CT had a good agreement with intraoperative findings in assessing intrahepatic vascular involvement by HAE (kappa index = 0.77). The estimated average ratio of RLV to SLV was 0.95 (range, 0.43–1.62). In conclusion, the 2D contrast-enhanced CT could well depict anatomic location and size of HAE, and invasion of porta hepatis and vascular by this disease, and involvement of other adjacent organs and tissues. Above all, 3D reconstruction could accurately measure RLV in patients with end-stage HAE for ALT.

## Introduction

Hepatic alveolar echinococcosis (HAE), caused by larval-stage echinococcus multilocularis, is a lethal parasitic disease and a near-cosmopolitan zoonosis^1^. The parasite often involves the major vessels in hepatic parenchyma and even invades or metastasizes to other organs like a malignant neoplasm^2–4^. Untreated HAE patients are estimated to have a 10-year mortality of 94%^5^. Radical hepatectomy has been considered as the best solution for HAE^6^. Due to the long asymptomatic incubation period, most of patients are at the advanced stage when diagnosed and seeking for treatment^7,8^, thus, cannot benefit from a radical resection. Autologous liver transplantation (ALT) is a recently introduced approach to cure end-stage HAE, which requires neither an organ donor nor any postoperative immunosuppressant^9,10^. However, ALT requires that hepatobiliary surgeons understand the invasion of intrahepatic structure and adjacent tissues or organs precisely. Therefore, preoperative meticulous assessment is of utmost importance.

Currently, triphase contrast-enhanced computed tomography (CT) of the liver has been widely accepted for diagnosis and preoperative evaluation of HAE due to its high scanning speed and high-density resolution^11^. With the careful evaluation at CT, the morphological characteristics of the lesions, the degree of vascular invasion, the invasion of adjacent tissues or organs, and even distant metastasis can be revealed. Three-dimensional (3D) reconstruction allows for intuitive and omnidirectional information of vascular and biliary anatomy, and accurate measurement of remnant liver volume (RLV) after virtual surgery. Up to now, most of the studies focus on the imaging diagnosis, the relationship between the lesion and intrahepatic vessels, and whether invasion of intrahepatic vessels or not^12–15^. There were no publications to report the utility of triphase enhanced CT together with 3D reconstruction in the comprehensive evaluation of preoperative indications for ALT in patients with end-stage HAE in detail. Therefore, the purpose of our study was to determine the value of triphase contrast-enhanced CT together with 3D reconstruction in preoperative assessments of indications for ALT.

## Materials and methods

### Ethical approval

The Institutional Review Board and Ethics Committee of the Affiliated Hospital of North Sichuan Medical College approved this study and waived patients’ informed consent because of nature of this retrospective study. All methods were performed in accordance with the relevant guidelines and regulations.

### Patients

From November 2016 to October 2020, 21 consecutive end-stage HAE patients who underwent preoperative CT followed by ALT were enrolled into our study. All patients received non-enhanced CT scans 3 to 5 days after ALT. There were 8 males and 13 females in our cohort with an average age of 30.5 years (range, 11–53 years). All patients' preoperative Child–Pugh classification of liver function was grade A. All patients received preoperative echinococcosis serological examination with positive results confirmed by postsurgical echinococcosis histopathological examination. The surgical indications for ALT in end-stage HAE patients included the following^9^: (1) HAE lesions invaded 2 or more crucial structures including the first, second and third porta hepatis; (2) retrohepatic vena cava (RHVC) was severely invaded and even obliterated with the appearance of ≥ 3 cm longitudinal and ≥ 180° circumferential invasion; (3) RHVC was severely invaded up to the pericardium/mediastinum/thoracic level; (4) patients had obstructive jaundice, but the serum total bilirubin level can be reduced to less than twice the upper limit of the normal value through percutaneous transhepatic cholangial drainage; (5) the ratio of RLV to standard liver volume (SLV) was estimated to more than 35%; and (6) patients had remote metastasis, but extrahepatic HAE lesions could be surgically removed or controlled by albendazole. The detailed important clinical information of each patient is recorded in Table 1. Of this cohort, distant metastasis was detected in 2 cases including one patient with HAE metastasis to right lung and the other with metastasis to retroperitoneal space, and both were controlled by albendazole. Additionally, statuses of porta hepatis and blood vessel invasion were confirmed by intraoperative observations and postoperative pathological examinations. In addition, 11 patients (52.4%) had postoperative complications including biliary leakage and liver dysfunction (Table 1). During the mean 29.0-month follow-up perriod (range, 3–47 months), all patients were alive.

**Table 1:** Clinical and CT data of each case.

Case	Sex	Age (y)	Location	LS (cm)	Porta hepatis involvement	RHVC invasion	LV (%)	RLV (ml)	SLV (ml)	RLV/SLV	PTLV (ml)	Adjacent organs and tissues involvement	Postoperative complication
1	M	25	RL and CL	12.7	First, second and third	9.4 cm, 360°	36.6	785.0	1095.5	0.72	944.7	Diaphragm	Yes
2	F	22	RL, CL and LML	16.7	First, second and third	12.4 cm, 360°	63.8	1080.5	1254.6	0.86	937.3	Diaphragm and right perirenal space	No
3	M	36	RL	17.4	Second and third	8.9 cm, ≥ 180°	58.1	1178.1	1173.6	1.00	987.3	Diaphragm and right adrenal gland	No
4	M	39	RL	14.9	Second and third	9.1 cm, ≥ 180°	50.0	1503.3	1321.7	1.14	1315.5	Diaphragm and right adrenal gland	No
5	M	28	RL	9.5	Second and third	6.5 cm, < 180°	27.9	1517.3	1276.9	1.19	1299.3	Diaphragm and right adrenal gland	No
6	F	22	RL, CL and LML	9.3	Second and third	8.2 cm, 360°	23.3	481.0	1121.9	0.43	416.2	Diaphragm and right adrenal gland	Yes
7	F	43	RL and CL	14.3	First, second and third	7.3 cm, ≥ 180°	38.3	1135.0	1238.1	0.92	1015.3	Diaphragm	No
8	F	27	RL	16.8	First, second and third	11.8 cm, 360°	49.1	1610.8	1082.9	1.49	1479.7	Diaphragm, right adrenal gland and gallbladder perihepatic space	Yes
9	F	34	RL and CL	12.1	First and third	6.4 cm, ≥ 180°	44.4	836.1	1025.0	0.82	743.3	Diaphragm, right adrenal gland and gallbladder	No
10	F	38	RL, CL and LML	11.6	First, second and third	8.4 cm, 360°	22.7	1162.1	1156.6	1.00	1016.0	Right adrenal gland	Yes
11	M	53	RL and CL	11.1	First, second and third	9.5 cm, 360°	40.1	885.2	1282.0	0.69	1053.2	Right diaphragm	No
12	M	18	RL and CL	15.7	First, second and third	5.4 cm, ≥ 180°	46.1	1803.6	1111.3	1.62	1744.0	Diaphragm and perihepatic space	Yes
13	F	11	RL and CL	9.9	Second and third	5.7 cm, ≥ 180°	15.5	990.3	1004.0	0.99	864.4	Right adrenal gland and perirenal space	Yes
14	F	45	RL	14.8	Second and third	8.6 cm, ≥ 180°	49.6	1117.2	1123.2	0.99	1076.7	Diaphragm and right adrenal gland	No
15	M	31	RL	16.6	First and third	4.5 cm, ≥ 180°	58.6	1047.6	1237.1	0.85	757.4	Diaphragm, right adrenal gland and gallbladder	No
16	F	26	RL	11.3	Second and third	5.6 cm, 360°	21.2	939.3	1191.7	0.79	1115.1	Right adrenal gland	Yes
17	F	29	RL	14.0	Second and third	10.4 cm, ≥ 180°	41.1	938.8	1039.6	0.90	945.9	Diaphragm and right adrenal gland	Yes
18	F	27	RL	10.8	Second and third	8.7 cm, ≥ 180°	26.8	1425.2	1200.8	1.19	1317.1	Diaphragm and right adrenal gland	Yes
19	M	24	RL and LML	20.2	First and third	6.5 cm, ≥ 180°	68.1	824.4	1215.8	0.68	765.7	Diaphragm and right adrenal gland	Yes
20	F	31	RL	13.0	No	7.6 cm, ≥ 180°	48.3	786.3	1080.5	0.73	642.6	Diaphragm and right adrenal gland	No
21	F	31	RL, CL and LML	11.6	First, second and third	7.1 cm, ≥ 180°	26.2	1039.8	1097.6	0.95	989.2	Diaphragm, right adrenal gland and gallbladder	Yes

*Notes* RL, right lobe; CL, caudate lobe; LML, left medial lobe; LS, lesion size; RHVC, retrohepatic vena cava; LV, lesion volume; RLV, remnant liver volume; and SLV, standard liver volume. In the column of RHVC invasion, the former values represent the longitudinal invasion and the latter represent the circumferential invasion.

### CT scans

Patients were fasted for at least 6 h before the preoperative CT examination, and drank 1000–1500 ml water 30 min to fill the gastrointestinal tract before scanning. A 128-detector CT scanner (Sensee 128; Siemens, Germany) was used to acquire precontrast scan and triphase contrast-enhanced CT scans including arterial phase, portal venous phase and delayed phase. The following scan parameters were used for the previous CT protocols: a peak tube voltage of 120 kV, automatic tube current modulation, rotation time of 0.5 s, collimation of 128 × 0.6 mm, a pitch of 0.6, a slice thickness of 10 mm, an interval of 10 mm, and a matrix of 512 × 512. The precontrast scan was performed to cover the whole liver. The subsequent tripple-phase scans were performed with the same scanning range after the intravenous injection of non-ionic iodinated contrast medium (Iodixanol; Beilu Beijing, China) into the right antecubital vein by a high-pressure injector (Ulrich, Germany) at an injection rate of 4 mL/s with a total dose of 75–150 ml for adults (90 ml for routine use) or of 2 ml/kg body weight for children, followed by a 20-mL saline solution flush. The arterial phase was acquired by performing a bolus tracking technique of the aorta. The scanning for arterial phase was automatically triggered 7 s after CT attenuation of the aorta at the level of the diaphragm had reached 100 Hounsfield Units. The scanning for the portal venous phase and delayed phase were set at 45 s and 75 s after the previous scanning for arterial phase, respectively. In addition, the scanning parameters for the postoperative CT scans were similar with those for the preoperative CT scans.

### Preoperative image analysis based on 2D CT data

The original preoperative CT data were reconstructed with a reconstruction slice thickness of 2 mm and an interval of 2 mm. Subsequently, the reconstructed data were sent to the workstation (sygno.via, Siemens, Germany) for the observation and measurement. The image analysis was carried out independently by two experienced radiologists (the first author and co first author with 6 and 11 years of experience in abdominal CT diagnosis, respectively), who were blinded to the laboratory data and clinical outcomes. Any discrepancies between the two observers were discussed until reaching a consensus.

**Figure 1:**
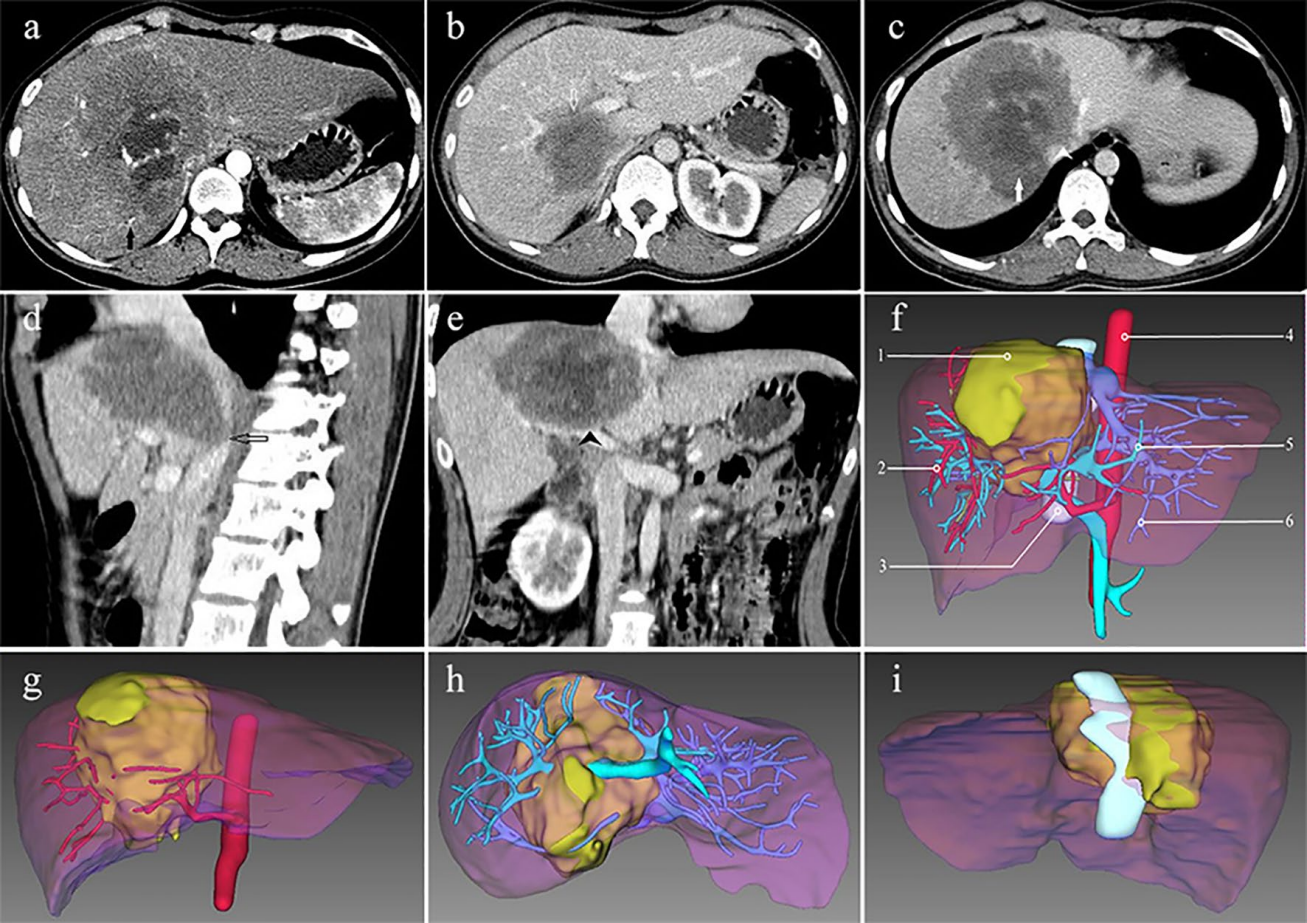
CT of a large hepatic alveolar echinococcosis located in the right liver lobe, caudate lobe and left medial lobe in case 21. Axial CT of the arterial phase (**a**) demonstrates that the right hepatic artery is of mild invasion by the lesion. Axial CT of the portal venous phase (**b**) displays the invasion of right portal vein with severe involvement (hollow arrow). Axial CT of the delayed phase (**c**) reveals obliteration of the right hepatic veins (arrow), and invasion of the second porta hepatis (arrowhead). Sagittal reconstruction of the portal venous phase data (**d**) displays severe invasion of retrohepatic vena cava (hollow arrow). Coronal reconstruction of the portal venous phase data (**e**) indicates the invasion of the first porta hepatis (arrowhead). Three-dimensional reconstruction image derived from the portal venous phase data (**f**) shows the location of the lesion and the relationship between the lesion and hepatic artery, portal vein, hepatic vein and inferior vena cava. The three-dimensional reconstruction images from arterial phase (**g**) and from the portal venous phase (**h** and **i**) data reflect the relationship between the lesion and hepatic artery, portal vein, hepatic vein and inferior vena cava in multiple-angle views, respectively. *Notes* 1, lesion; 2, hepatic artery; 3, inferior vena cava; 4, abdominal aorta; 5, portal vein; and 6, hepatic vein.

Basic imaging manifestations associate with the HAE lesions, including the size and location of the lesions, and the invasion of hepatic hilum and adjacent organs or tissues, were obtained from the data of triphase enhanced CT. The measurement of lesion size and evaluation of adjacent organs or tissues invasion were performed in portal venous phase images because the profile of the HAE lesion could be depicted well in this phase. Arterial phase and portal venous phase images were applied to evaluate the invasion of first hepatic hilum by HAE, and delayed phase images were used to evaluate the invasion of second and third hepatic hilum. In addition, post processing technologies including maximum intensity projection (MIP), volume rendering (VR) and multiplanar reconstruction (MPR) were performed in the arterial phase, portal vein phase and delayed phase to evaluate the invasion extents of intrahepatic vessels including hepatic artery, portal vein and hepatic vein, respectively (Fig. 1a–e).

### 3D reconstruction technology

The preoperative CT data were loaded onto the 3D reconstruction software (IQQA-Liver, EDDA Technology, Princeton, NJ, USA) to reconstruct the 3D model of the diseased liver. Based on the preoperative triphase enhanced images, important structures including the hepatic vein (HV), RHVC, portal vein (PV) and hepatic artery (HA) were extracted from CT data by segmentation and were used to generate 3D model. The 3D reconstruction model could be amplified, rotated, and hyalinized to clarify the anatomic character of tissue structure and the relations between the lesion and peripheral tissues with omnidirectional, multiple-angle and multilevel views (Fig. 1f–i).

Above all, the 3D model of the diseased liver was used to measure the volume of the whole liver and HAE lesions in all patients. Firstly, the portal venous phase images were imported into the software, which could automatically generated 3D liver model and calculated the total liver volume (TLV). Subsequently, observer manually outlined the maximum diameter of the HAE lesion based on the density difference between the lesion and normal liver parenchyma, the boundary of the lesion could be identified automatically by the software to reconstruct 3D model of the lesion and calculate the lesion volume (LV). Virtual surgery was performed on the reconstructed 3D model and the RLV was calculated automatically. This measurement was performed in cooperation with a radiologist and a hepatobiliary surgeon. All the images were corrected layer by layer to obtain the accurate boundary of the lesion and the liver. In addition, SLV estimation was based on the formula in consideration of body surface area (BSA)^16^: SLV = 706.2 × BSA + 2.4, where BSA was calculated in consideration of height in centimeter and body weight (BW) in kilogram using the Dubois formula: BSA = 0.007184 × Height^0.725^ × BW^0.4253^. Ultimately, the ratio of RLV to SLV (RLV/SLV) could be obtained. Additionally, the post-surgical total liver volume (PTLV) was also obtained by the method similar with TLV measurement on the postoperative non-enhanced CT to confirm the accuracy of the previous RLV measurement.

In order to show the benefit of the previous 3D reconstruction for RLV measurement, we performed the comparative study on the accuracy of 3D vs. 2D methods to measure RLV. The portal venous phase images were selected to measure the RLV with 2D method by a radiologist and a surgeon working in consensus on an image-processing workstation (sygno.via, Siemens, Germany). After the surgeon outlined the surgical resection range on the CT image, the remnant liver profile was subsequently determined, and then the remnant liver was recorded for every two slices from the superior aspect to the lower aspect of the liver. The area of the remnant liver was measured slice by slice, and the RLV were estimated by using the computing function of the workstation.

### Degree of hepatic vascular invasion

Based on the degrees of circumferential invasion of HV, RHVC, PV and HA, the cross-sectional area of the remained lumen due to vascular stenosis secondary to the vascular invasion, the status of collateral circulation as shown on preoperative triphase contrast-enhanced CT, and the degrees of hepatic vascular invasion were classified as mild, moderate and severe invasion. The degree of the vessel invasion was defined as mild invasion with the vessel of < 180° circumferential invasion, and at least half of lumen cross-sectional area remained with normal blood supply or without collateral circulation. The degree of invasion was defined as moderate invasion if the vessel was of ≥ 180° and < 270° circumferential invasion, and less than half of lumen cross-sectional area was remained with or without the formation of collateral circulation. The degree of invasion was defined as severe invasion when the vessel was of ≥ 270° circumferential invasion, and lumen cross-sectional area almost disappeared together with the formation of collateral circulation. The intraoperative evaluation of the degree of hepatic vascular invasion was also based on the previous CT definition. The above-mentioned diagnostic criteria of the degree of vascular invasion were based on the different surgical treatment according to the degree of vascular invasion. For the mild invasion, the patients would not receive special treatment, or would undergo local remove of blood vessel. For the moderate invasion, at least half circumferential vascular wall should be removed, and then the vascular patch would be used to repair the previously removed vascular wall. For the severe invasion, the entire circumferential vascular wall was removed, and subsequently was reconstructed or replaced completely.

### Statistical analysis

The analysis was performed with a statistical software package (version 13.0 for Windows, SPSS Inc, Chicago, IL, USA). Chi-squared test or Fisher’s exact test were used to compare the incidences of porta hepatis invasion and intrahepatic vascular invasion between groups of HAE lesions located in the right liver lobe (group 1) and in both the right liver lobe and the caudate lobe / left medial lobe (group 2). These tests were also used to compare the incidences of individual vessel involvements at the moderate to severe degrees of vascular invasion and the incidences of postoperative complication of the patients stratified by the ratio of RLV to SLV. A *P* value < 0.05 indicated to be significant. In addition, all measured parameters such as RLV were expressed as the average from the two observers.

The Cohen’s kappa-coefficient test was employed to measure the agreement between the preoperative imaging diagnosis and intraoperative diagnosis for assessing the vascular invasion by HAE. A kappa-coefficient of < 0.01, 0.01–0.20, 0.21–0.40, 0.41–0.60, 0.61–0.80 and 0.81–1.00 indicated no agreement, poor agreement, mild agreement, acceptable agreement, good agreement and almost perfect agreement, respectively. In addition, the agreements between the RLV measurement by 3D or 2D method, and the PTLV measurement was performed by using the interclass correlation coefficient (ICC). The agreement was defined as excellent (ICC > 0.90), good (ICC = 0.75–0.90), moderate (ICC = 0.5–0.75), or poor (ICC < 0.5)^17^.

## Results

### General characteristics

In this cohort, a total of 10 (47.6%), 6 (28.6%), 1 (4.8%), and 4 (19.0%) patients had lesions involving the right liver lobe, both the right and caudate liver lobes, both the right and left medial liver lobes, and the right and caudate and left medial liver lobes, respectively. The mean value of maximum diameter of lesions was 13.5 cm (range, 9.3–20.2), and the corresponding average proportion of LV in TLV was 40.8% (range, 15.5%-68.1%). The adjacent organs and tissues involved by HAE included diaphragm (85.7%, 18/21), right adrenal gland (76.2%, 16/21), gallbladder (19.0%, 4/21), perirenal space (9.5%, 2/21), and perihepatic space (4.8%, 1/21). The detailed CT characteristics of each HAE are listed in Table 1.

### Porta hepatis invasion

This cohort comprised 20 patients (95.2%, 20/21) with porta hepatis invasion and one patient without porta hepatis invasion (Table 1). HAE lesions were located in the right liver lobe in 10 patients (group 1) and in both the right liver lobe and the caudate lobe / left medial lobe in 11 patients (group 2). In group 1, porta hepatis invasion occurred most commonly in the second and third porta hepatis in 70.0% patients (7/10), whereas the first, second and third porta hepatis were most commonly involved in group 2 in 63.6% patients (7/11) with significant difference (*P* = 0.015).

### Vascular invasion

Of the 21 patients with RHVC invasion as shown in Table 1. The mean value of longitudinal invasion was 8.0 cm (range, 4.5–12.4). In this cohort, 95.2% (20/21) of them had RHVC invasion with the appearance of ≥ 180° circumferential invasion and 4.8% (1/21) of them had the appearance of < 180° circumferential RHVC invasion. There were no patients with RHVC invasion up to the pericardium, mediastinum, and/or thoracic level.

The degree of intrahepatic vascular involvement as shown in preoperative imaging is described in Table 2. Each patient had 8 intrahepatic vessels including HV (left, middle and right), RHVC, PV (left and right branch), and HA (left and right). In group 1, 15.0% (12/80), 11.3% (9/80) and 40.0% (32/80) of vessels had mild, moderate and severe invasion, respectively; and 33.7% (27/80) of vessels had no invasion. Of the 88 intrahepatic vessels in group 2, the mild, moderate and severe vessel invasion were found in 23.8% (21/88), 9.1% (8/88) and 48.9% (43/88) vessels, respectively, and 18.2% (16/88) of vessels had no invasion. Intrahepatic vessels involvement were more frequently seen in the group 2 than in the group 1 (*P* = 0.021). However, there were no significant differences in the degrees of intrahepatic vascular invasion between two groups (*P* > 0.05). Of all the 21 patients, moderate and severe invasion occurred most commonly in the right hepatic vein, right branch of portal vein and RHVC each in 20 (95.2%) patients, followed by that in middle hepatic vein in 66.7% (14/21) patients and in right hepatic artery in 61.9% (13/21) patients (both *P*-values < 0.05).

**Table 2:** The preoperative evaluation of the degree of intrahepatic vascular invasion in all the 21 patients.

Vessel in groups	Non	Degree of invasion		
		Mild	Moderate	Severe
**Group 1 (n = 80)**				
LHV	9	0	1	0
MHV	3	2	3	2
RHV	0	0	0	10
RHVC	0	1	3	6
LPV	5	5	0	0
RPV	0	0	1	9
LHA	10	0	0	0
RHA	0	4	1	5
**Group 2 (n = 88)**				
LHV	5	4	1	1
MHV	1	1	1	8
RHV	1	0	1	9
RHVC	0	0	2	9
LPV	2	7	2	0
RPV	1	0	0	10
LHA	6	5	0	0
RHA	0	4	1	6

*Notes* LHV, left hepatic vein; MHV, middle hepatic vein; RHV, right hepatic vein; RHVC, retrohepatic vena cava; LPV, left portal vein; RPV, right portal vein; LHA, left hepatic artery; and RHA, right hepatic artery.

In addition, with regard to the results from the comparisons of the preoperative CT diagnosis for assessing the intrahepatic vascular involvement by HAE and the intraoperative diagnosis as depicted in Table 3, its kappa index was 0.77.

**Table 3:** Preoperative imaging diagnosis and intraoperative diagnosis of vascular invasion in all the 21 patients.

Preoperative imaging diagnosis (n = 168)	Intraoperative diagnosis (n = 168)			
	Non	Mild	Moderate	Severe
Non	42	1	0	0
Mild	1	25	5	2
Moderate	0	4	1	12
Severe	0	0	1	74

*Notes* Each patient has 8 intrahepatic vessels in total, and then all patients in our cohort have 168 vessels.

### Remnant liver volume

The average RLV measured by the 3D and 2D methods were 1099.4 ± 320.4 and 942.5 ± 286.5 mL, respectively. The ICC values between PTLV and the RLV estimated by 3D or 2D methods were 0.921 or 0.777, respectively. Because the ICC value between PTLV and the RLV estimated by 3D method was higher than that between PTLV and the RLV estimated by 2D method, we performed the subsequent study based on the RLV estimated by 3D method.

The mean value of the estimated RLV by 3D method was 1099.4 mL (range, 481.0–1803.6), PTLV was 1020.3 mL (range, 416.2–1744.0) and the corresponding average RLV/SLV was 0.95 (range, 0.43–1.62). In addition, this cohort was divided into three groups based on the ratio of RLV to SLV. In detail, when the ratios of RLV to SLV were 0.35–0.75, 0.75–1.0 and > 1.00, the corresponding incidences of postoperative complications was 60%, 45.5% and 60%, respectively (Table 1). There was no statistical significance in the incidence of postoperative complications between groups stratified by the ratio of RLV to SLV (*P* > 0.05).

## Discussion

HAE exhibits an invasive growth and tends to occupy the hepatic parenchyma and invade intrahepatic vessels in particular^4,12^. Unlike malignant tumors, a relatively long time interval between primary infection and presentation of symptoms enables adequate compensatory function of the disease-free lobe of the liver in end-stage HAE patients. For patients with end-stage HAE that are otherwise unresectable by conventional procedures, ALT may offer reasonable outcomes due to their sufficient graft volumes (disease-free lobe)^4,10^. Since this challenging technique was first reported by Pichlmayr and colleagues in 1988^18^, Wen and Yang successfully treated dozens of cases with end-stage HAE with this surgical procedure^4,9^. Because ALT is a complex and radical procedure, an accurate and comprehensive preoperative assessment of ALT indications is important for successful surgery^4,9,13,19^. In this study, we aimed to investigate the feasibility of CT together with 3D reconstruction to preoperatively evaluate the indications for ALT in patients with end-stage HAE.

The 2D triphase contrast-enhanced CT could demonstrate the location and size of HAE lesions, and the invasion of the porta hepatis, intrahepatic vessels and the adjacent structures for ALT. 3D reconstruction of a HAE lesion and the liver intuitively reflects the location of the lesion, and the relationship between the lesion and intrahepatic vessels, which could provide a visual assessment for hepatobiliary surgeon to plan operation scheme. Most important of all, the 3D reconstruction system allows surgeons to perform virtual surgeries on the 3D model of a liver to obtain the accurate RLV, which is a key factor of surgical indications^4,9,13^.

Our study revealed that the invasion rate of porta hepatis could be highest in the second and third porta hepatis in patients with HAE located in right hepatic lobe, while the first, second and third porta hepatis could be involved in patients with the lesions in right and caudate / left medial hepatic lobes. The result could be explained by the fact that the lesions in the right liver lobe involving the caudate and left medial lobes can be closer to the first porta hepatis than those in the right lobe. Lesions in the right hepatic lobe can grow along the RHVC and invade the second and third porta hepatis directly. As the lesion grows to involve caudate / left medial lobes, the first porta hepatis is surrounded by the lesion. Therefore, patients with HAE lesions in the right and caudate / left medial lobes can be more likely to invade the first porta hepatis besides the second and third porta hepatis. This finding can help clinicians predict the feasibility of porta hepatis invasion by HAE to guide the treatment options.

Based on the contrast-enhanced CT, we found that moderate to severe invasion of intrahepatic vessels could occur most commonly in the right hepatic vein, right branch of portal vein and RHVC. The possible reasons are as follows. Since all lesions involved the right liver lobe in this study, the intrahepatic vessels of the right lobe tended to be invaded more frequently. When humans are infected with echinococcus multilocularis by ingestion of contaminated food and water, oncospheres hatch from eggs in the small intestine, and migrate to the liver via the portal system, where they invade the liver tissues and vasculature and metastasize to any other organ via hepatic venous drainage into the inferior vena cava. The infection and transmission route are intimately associated with the portal venous invasion and venous invasion^20^. Veins are thicker than arteries in the diameters of blood vessel at the cross section and have more blood flow, suggesting that veins are more likely to be invaded than arteries.

Our intraoperative diagnosis allows the preoperative triphase contrast-enhanced CT evaluation of the degree of intrahepatic vascular invasion. We found a good agreement between preoperative imaging diagnosis and intraoperative diagnosis in assessing the degree of intrahepatic vascular involvement in patients with end-stage HAE. Supporting this estimate, Frikha et al.^14^ reported that the agreement between CT and surgery is excellent in the study of the relationship between hepatic hydatid cyst and RHVC, HV and HPV. The difference in our study from this previous report is that we first confirmed triphase enhanced CT could depict the degree of intrahepatic vascular invasion by HAE for identifying the indications of ALT whereas the published study focused on the feasibility but not on the degree of intrahepatic vascular invasion.

Clinical experience has demonstrated that the graft liver with a small-for-size is prone to complications and liver failure^10^. In planning ALT, the RLV should be precisely measured to assess the indications of this surgery in order to prevent the occurrence of small-liver syndrome^21^. Our study showed that the agreement between the RLV measurement by 3D method and the PTLV measurement was excellent when compared with the traditional 2D measurement method (ICC: 0.921 vs. 0.777), suggesting the preoperative 3D reconstruction by virtual surgery can be a good procedure to measure RLV.

In this study, the estimated ratio of RLV to SLV of all patients was larger than 35%, which suggested that the RLV was adequate to meet the functional demands. ALT was successful in all patients without intraoperative mortality and with 100% survival during the follow-up period, indicating that there was no difference in patients’ survival rate if the ratio of RLV to SLV was larger than 35%. In addition, our study revealed that there was no difference in the incidences of postoperative complications between groups stratified by the ratio of RLV to SLV. We considered that RLV could not be associated with postoperative complication.

### Limitations

This study has inevitable certain shortcomings. The first limitation is insufficiency of swatches and lack of a control group. The second limitation is that we did not explore in which way CT helped in planning surgery, and we will perform the relevant study in the future. The third limitation is a pitch factor of 0.6 used in the CT scanning protocol. We will perform our future study by using a higher pitch factor to reduce the radiation exposure to confirm our findings. The fourth limitation is that we did not compare CT with any other technique for the assessment of HAE lesions, and compare CT with and without 3D reconstruction to assists optimizing ALT in vascular infiltration. We will perform the relevant study in the future to show how to optimize ALT by CT with 3D reconstruction.

## Conclusion

The application of triphase enhanced CT together with 3D reconstruction could help evaluate important indications in patients with end-stage HAE for ALT. In detail, the 2D contrast-enhanced CT could well depict anatomic location and size of HAE, and invasion of porta hepatis and vascular by this disease, and involvement of other adjacent organs and tissues. Above all, 3D reconstruction could accurately measure RLV/SLV in patients with end-stage HAE for ALT. We believe that our findings could help well depict the indications of ALT and increase the chance of success of this surgery.
